# Trends and hotspots in research related to tumor immune escape: bibliometric analysis and future perspectives

**DOI:** 10.3389/fimmu.2025.1604216

**Published:** 2025-08-28

**Authors:** Houcheng Zhu, Yue Huang, Xiangjin Wang, Wang Xiang, Yong Xie

**Affiliations:** ^1^ School of Sports Medicine and Health, Chengdu Sports University, Chengdu, China; ^2^ Hospital of Chengdu University of Traditional Chinese Medicine, Chengdu, China

**Keywords:** tumor immune escape, bibliometric analysis, tumors, tumor microenvironments, immune checkpoint inhibitors

## Abstract

**Background:**

Tumor immune escape, a defining hallmark of malignant tumors, enables cancer cells to thrive within the host by evading detection and attack by the immune system. While immune checkpoint inhibitors, such as PD-1/PD-L1 antibodies, have delivered significant clinical advances, their effectiveness is tempered by modest response rates and a growing challenge of drug resistance. In this study, we aimed to explore the development process and trend of tumor immune escape, analyze the current hot spots, and predict the future research directions.

**Methods:**

A bibliometric analysis was conducted in this study to retrieve and analyze 1839 publications from January 1, 2009 to February 14, 2025 related to tumor immune escape. Literature was obtained from Web of Science Core Collection (WoSCC) and data visualization and trend analysis were performed using VOSviewer, CiteSpace, Bibliometrix software package.

**Results:**

The bibliometric analysis indicates that research on tumor immune escape has primarily focused on China, the United States, and European countries. China ranks first in research output and impact, with notable contributions from institutions like the Sun Yat-sen University System and the University of Texas System. The journal with the most publications is Frontiers in Immunology, while the most cited article globally is Jiang P’s 2018 publication in Nature Medicine, titled “Signatures of T cell dysfunction and exclusion predict cancer immunotherapy response.” Keyword co-occurrence and burst analysis indicate that the field has undergone a thematic evolution. Early research centered around classical immune checkpoint molecules and T cell exhaustion, while more recent trends have shifted toward the tumor microenvironment (TME), multi-target combination immunotherapies, and mechanisms of immune evasion involving metabolic reprogramming and the microbiome. The integration of artificial intelligence (AI) and machine learning (ML) in immunotherapy prediction and biomarker discovery has also gained momentum, highlighting a growing cross-disciplinary approach.

**Conclusion:**

This bibliometric study provides a comprehensive overview of the intellectual landscape, research hotspots, and developmental trajectory of tumor immune escape research over the past 14 years. By mapping influential nation, authors, core journals, reference, and keyword bursts, this work not only summarizes major contributions in the field but also helps researchers better understand its evolution and emerging directions. Based on the observed patterns, we propose three key areas that warrant further exploration: (1) advancing interdisciplinary research at the intersection of the microbiome, metabolism, and immune regulation; (2) integrating artificial intelligence and multi-omics data to enhance predictive modeling and therapeutic precision; and (3) combining multi-modal therapeutic strategies to overcome immune escape more effectively.

## 1 Introduction

Cancer has become a globally prevalent and serious economic and social problem, with increasing incidence and high mortality rates (1). Although the traditional three main therapies (surgery, radiotherapy, and chemotherapy) remain the cornerstone of clinical treatment, their efficacy is limited by significant toxicities and patient response heterogeneity. Targeted therapies have achieved significant breakthroughs in treating specific malignancies by blocking key oncogenic signaling pathways, such as those involving the epidermal growth factor receptor, HER2, estrogen receptor, vascular endothelial growth factor recepto, and multikinase inhibitors (2). However, issues of acquired drug resistance and inadequate therapeutic efficacy remain unresolved, particularly in cancers with complex pathophysiologic mechanisms.

The advent of cancer immunotherapies has revolutionized treatment approaches, particularly with immune checkpoint inhibitors like anti-PD-1/PD-L1 antibodies marking a landmark advancement. This breakthrough, awarded the 2018 Nobel Prize in Physiology or Medicine, combats tumor immune evasion by enhancing T-cell-mediated antitumor immune responses (3). However, despite these advances, not all patients benefit clinically due to the dynamic complexity and spatial heterogeneity of TME (4). Recent studies have demonstrated that malignant cells employ various strategies to create immune-evasive microenvironments, including metabolic reprogramming, secretion of immunosuppressive factors, and epigenetic modulation of antigen-presentation mechanisms (5, 6).

Tumor immune escape is a phenomenon where tumor cells avoid immune system recognition and attack, enabling them to grow and metastasize. This is a key strategy for tumor survival and progression (7). The interaction between immunity and cancer in regulating tumor growth is considered a cancer hallmark. Anti-tumor immunity involves innate and adaptive immune responses that control cancer development and proliferation. Tumor immune escape poses a major obstacle to effective anticancer therapy (8). Many factors induce tumor immune escape, including low tumor cell immunogenicity, tumor-specific antibody recognition as self-antigens, tumor surface antigen regulation, tumor-induced immune privilege, and tumor-induced immunosuppression. Research mainly focuses on the latter factors. Cancer cells can evade the immune system by activating immune checkpoints, altering the surrounding microenvironment, causing antigen presentation and recognition abnormalities, and undergoing metabolic reprogramming to inhibit T-cell activity. This allows cancer cells to survive and proliferate within the host (9). This mechanism is significantly influenced by programmed death receptor 1/programmed death receptor-ligand 1 (PD-1/PD-L1), which regulates immune tolerance and escape within TME (7, 10–12). When the PD-1 receptor on activated T cells interacts with the PD-L1 receptor on cancer cells, it weakens cytotoxic T lymphocyte effects, helping malignant cells resist immune attacks and promoting immune escape (13).

In recent years, research into tumor immune escape mechanisms and their role in cancer progression has surged exponentially. Reviews have explored key issues in this field from molecular pathways and clinical interventions perspectives (4, 14). However, a systematic overview of the discipline’s development and knowledge structure evolution is still lacking, as is a clear definition of research foci and potential blind spots. Bibliometrics, an effective tool for assessing discipline dynamics, can objectively identify core contributing countries, institutions, and scholars, and reveal landmark high-impact literature. It can also track historical changes in research hotspots, capture emerging frontier directions, and locate under - explored scientific issues (15). Such analyses have been successfully applied to TME (16), checkpoint inhibitor development (17) and other immunotherapy - related fields. Notably, while bibliometric studies on the PD-1/PD-L1 signaling axis or CAR-T cell therapies have been reported (18), there remains a lack of comprehensive and systematic analyses specifically focused on the field of tumor immune escape. Therefore, this study aims to comprehensively analyze the research landscape, evolutionary pathways, and future trends in tumor immune escape using a multidimensional bibliometric approach. This will provide a data - driven decision - making basis for optimizing immunotherapeutic strategies and basic research directions.

## 2 Materials and methods

### 2.1 Data collection and sources

Bibliometric analysis offers a systematic framework for identifying developmental trends and research hotspots within a discipline over a defined period. The selection of an appropriate database is critical to ensuring data reliability and analytical rigor. Among available options, the Web of Science (WoS) stands out for its multidisciplinary coverage of high-impact scientific journals and robust citation indexing. Compared to Scopus and MEDLINE/PubMed, WoS provides more comprehensive information that is particularly well-suited for bibliometric analysis (17). In this study, we selected the Web of Science Core Collection as our primary data source, as it is widely recognized for its depth, accuracy, and authority in indexing peer-reviewed literature (19). Its extensive journal coverage ensures that the retrieved publications reflect contemporary research trajectories in immunology and oncology. This choice enables accurate, representative data extraction and supports a thorough exploration of valuable research insights.

### 2.2 Search strategy and criteria

**Figure 1:**
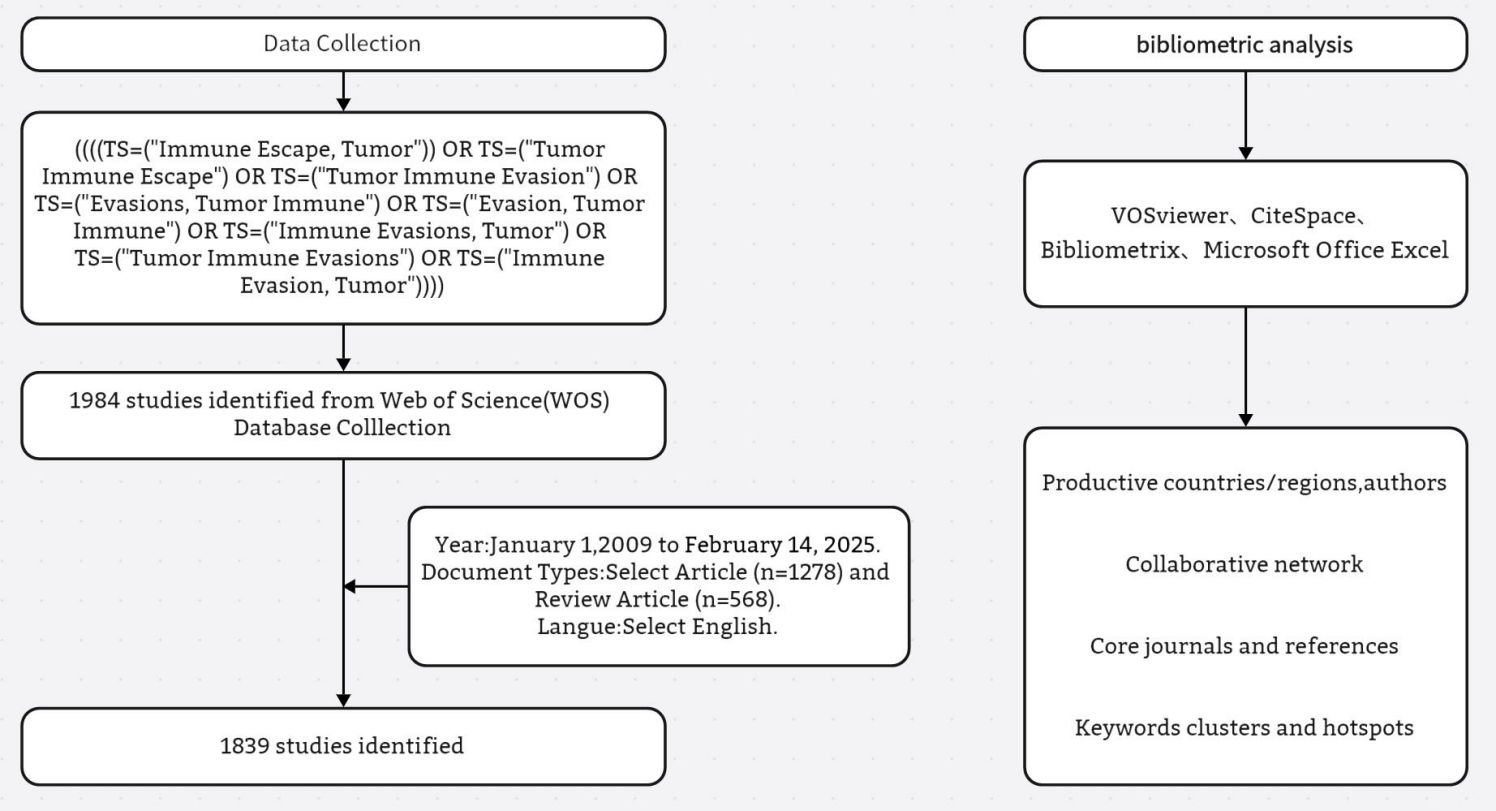
A process flowchart.

A literature search was conducted on February 14, 2025, to retrieve original articles and reviews on tumor immune escape published between 2009 and February 14, 2025. To avoid temporal bias due to real-time database updates, the search was completed in a single day. The search strategy was as follows: ((((TS=(“Immune Escape, Tumor”)) OR TS=(“Tumor Immune Escape”) OR TS=(“Tumor Immune Evasion”) OR TS=(“Evasions, Tumor Immune”) OR TS=(“Evasion, Tumor Immune”) OR TS=(“Immune Evasions, Tumor”) OR TS=(“Tumor Immune Evasions”) OR TS=(“Immune Evasion, Tumor”)))). Only journal articles and reviews published in English were included in this analysis. Other publication types—such as letters, editorials, conference abstracts, meeting reports—and all non-English publications were excluded to ensure consistency and comparability of the bibliometric dataset. The eligible records were exported in plain-text format with the “Full Record and Cited References” option selected to enable comprehensive metadata extraction. The final dataset contained information on publication counts, citations, titles, authors, affiliations, countries, keywords, and journals. In total, 1,839 records met the inclusion criteria. The detailed screening process is presented in **Figure 1**.

### 2.3 Data analysis

For data processing and analysis, we used Microsoft Excel in combination with three specialized tools: Bibliometrix 4.3.3 (an R-based package), VOSviewer 1.6.20, and CiteSpace 6.4.R1.

VOSviewer, developed by van Eck and Waltman, generates bibliometric network visualizations using node-link diagrams. It visualizes collaboration patterns by clustering nodes chromatically, where node size represents publication volume and edge thickness indicates collaboration strength between entities (e.g., countries or institutions).

CiteSpace, created by Chaomei Chen, is a Java-based software for detecting research frontiers. It employs timeline mapping and citation burst detection, with keyword clustering to reveal thematic domains. Clustering reliability is validated when silhouette values exceed 0.5 and modularity Q-values exceed 0.3, indicating strong internal consistency and significant structural separation.

Bibliometrix, an R-integrated package, enables statistical analysis of scholarly outputs including publication frequencies, citation metrics, and national contributions. Its algorithms support cross-comparison among journals and countries, contributing to a quantitative understanding of academic productivity.

## 3 Result

### 3.1 Trends in publications and citations

**Figure 2:**
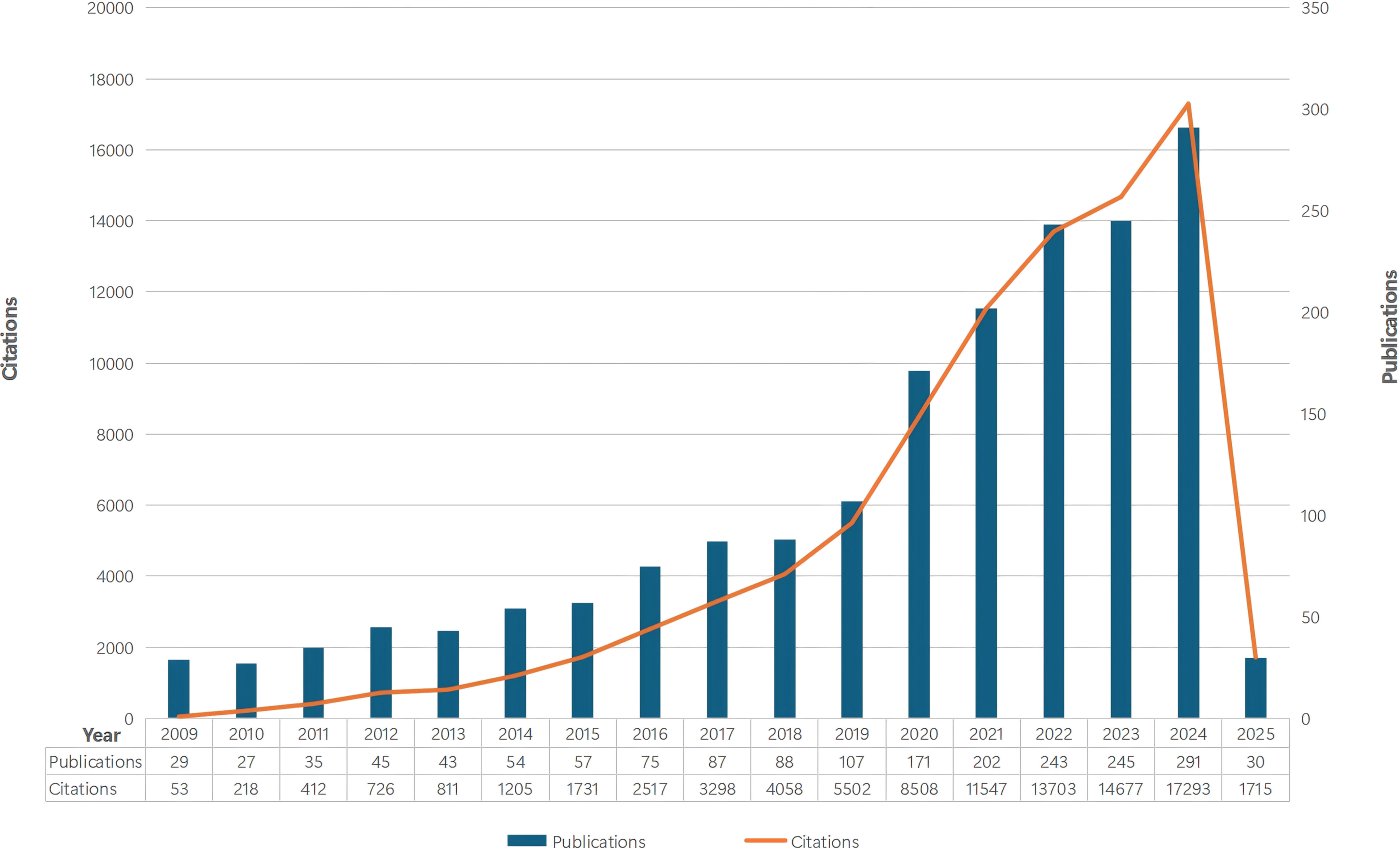
The number of annual papers and citations on tumor immune escape research has been steadily increasing from 2009 to 2025.

As per the formulated research strategy, 1,839 tumor immune escape - related publications were obtained from the WoSCC database between 2009 and 14 February 2025. **Figure 2** presents the annual publication and citation counts for tumor immune escape research from 2009 to 14 February 2025.

The steady publication increase from 2009 to 2018 shows great attention and interest in this field. The steeper growth curve from 2018 onwards indicates significant expansion, likely due to the 2018 Nobel Prize in Physiology or Medicine awarded to Professors James P. Allison and Tasuku Honjo for their work on the CTLA - 4 and PD - 1/PD - L1 pathways. The rising citation trend suggests ongoing research impact and the need for more prospective studies to highlight its global relevance.

### 3.2 National and institutional analyses

**Figure 3:**
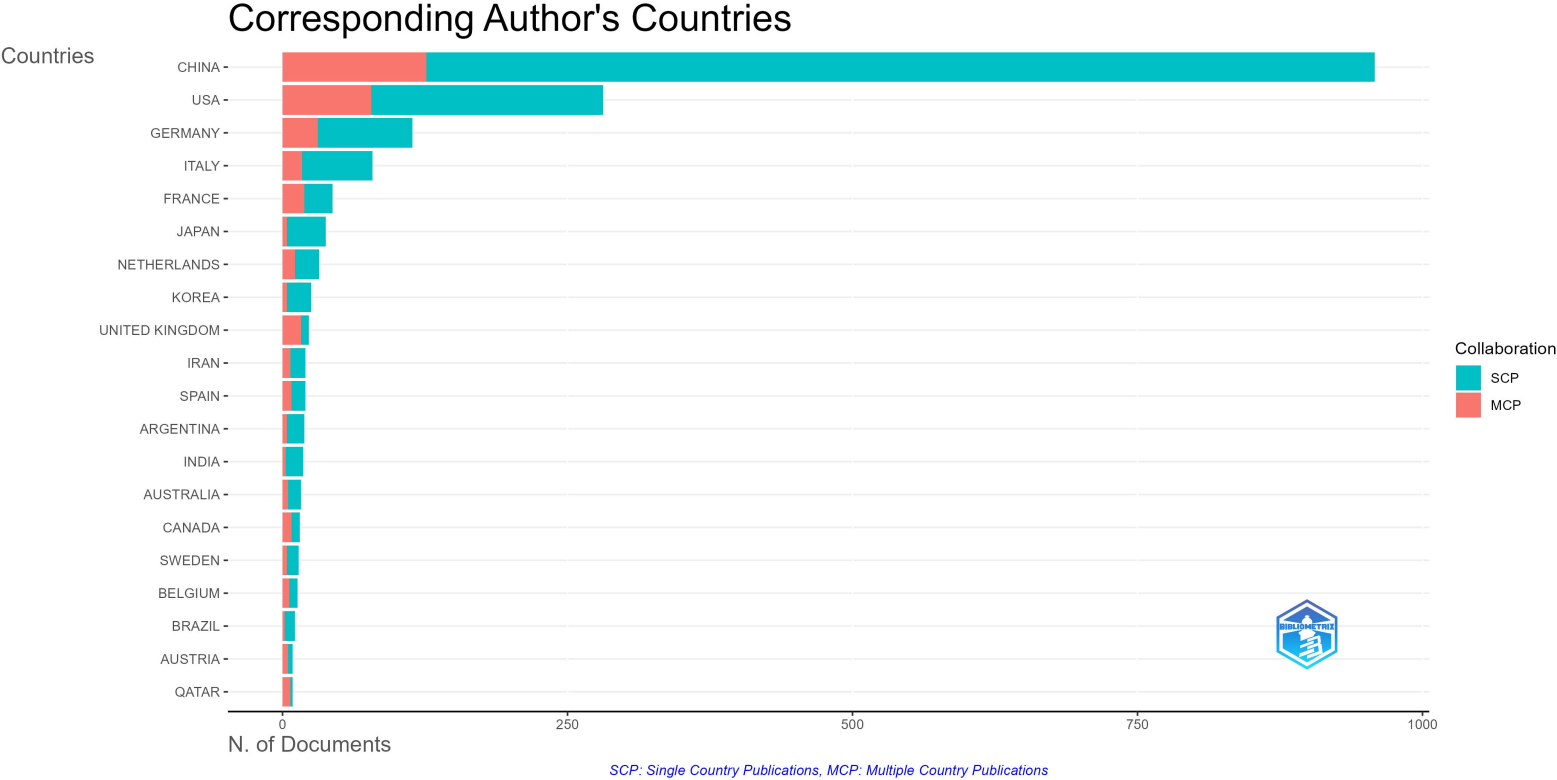
Top 20 most corresponding author’s country in the tumor immune escape field.

A total of 65 countries and 2,184 institutions participated in tumor immune escape research. **Table 1** ranks the top ten countries by number of publications and total citations. China (n = 958) was the most productive country, accounting for 52.1 per cent of the total number of publications, followed by the United States (n = 281, 15.3 per cent), and Germany (n = 114, 6.2 per cent). The US and China have nearly identical total citations, 28,052 and 28,142 respectively, far surpassing other nations and highlighting their influence in this field. The UK has the highest average citations per publication at 131. Multiple country publication (**Figure 3**) refers to the proportion of publications in this field involving contributions from multiple countries and is used to assess international collaboration levels within a research area in a given country. China has the highest number of publications, but the proportion of multiple country publication with other countries is relatively low at 13.2%. However, France (43.2%) and the UK (69.6%) have a high proportion of multiple country publication, indicating their significant contributions to international cooperation.

**Table 1:** Top 10 productive countries of publications on tumor immune escape.

Rank	Country	Articles n(%)	SCP	MCP	MCP %	Country	TC	AC
1	CHINA	958 (52.1%)	832	126	13.2	CHINA	28142	29.40
2	USA	281 (15.3%)	203	78	27.8	USA	28052	99.80
3	GERMANY	114 (6.2%)	83	31	27.2	GERMANY	6284	55.10
4	ITALY	79 (4.3%)	62	17	21.5	ITALY	3270	41.40
5	FRANCE	44 (2.4%)	25	19	43.2	UNITED KINGDOM	3013	131.00
6	JAPAN	38 (2.1%)	34	4	10.5	SPAIN	2154	107.70
7	NETHERLANDS	32 (1.7%)	21	11	34.4	NETHERLANDS	1842	57.60
8	KOREA	25 (1.4%)	21	4	16	FRANCE	1783	40.50
9	UNITED KINGDOM	23 (1.3%)	7	16	69.6	CANADA	1647	109.80
10	IRAN	20 (1.1%)	13	7	35	JAPAN	1631	42.90

NP, number of publications; SCP, single country publication; MCP, multiple country publication; TC, total citation; AC, average citations.

**Figure 4:**
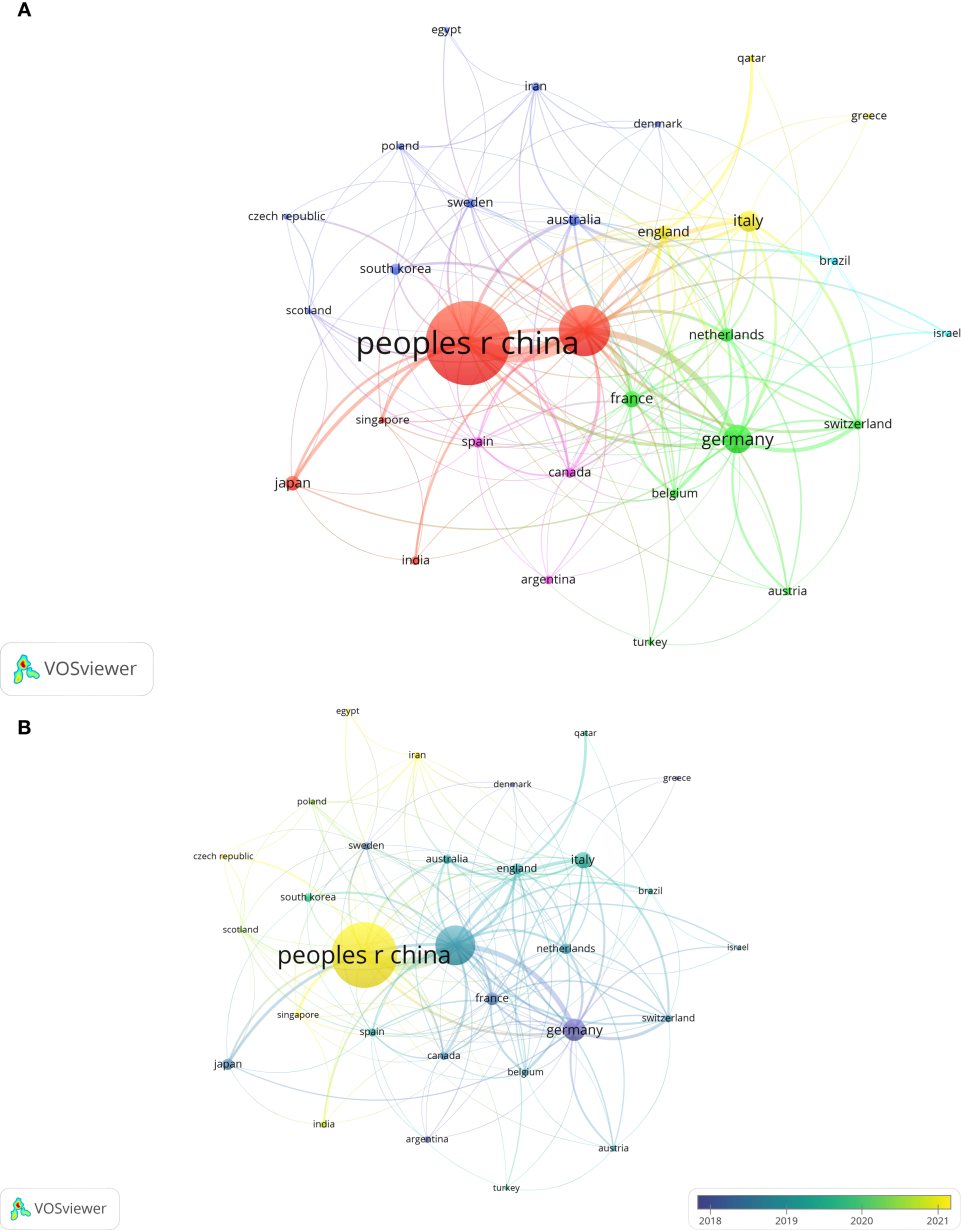
**(A)** Visual map of national/regional citation networks. Each circle/node’s size shows the number of papers published. The connection strength between circles/nodes is indicated by the line thickness, with colors representing clusters of related objects in the network. Each circle/node stands for a separate country/region. **(B)** Visualisation map of the country/region citation overlay. Purple nodes are organisations that started research in this area earlier, while yellow nodes are those that began later.

A minimum threshold of 7 articles was set to filter out 30 countries meeting the criteria, as shown in **Figures 4A, B**. This reveals a wide - ranging network of international cooperation, with the US, China, and various European countries serving as key hubs. The United States led international collaboration with the highest total link strength at 303, underscoring its central role in the global tumor immune-escape network. China followed at 204, and Germany at 117, together highlighting these nations’ pivotal contributions to cross-border research and knowledge exchange in the field. Notably, the closest collaboration exists between the US and China. Additionally, publication timelines were analyzed through a VOSviewer - based visual map of organizational collaboration overlays (**Figure 4B**). It is worth noting that China started publishing later than most other leading countries in this field.

Among the top 15 institutions ranked by publication count (**Table 2**), Sun Yat-sen University leads with 72 publications, followed by the Chinese Academy of Sciences with 69. This indicates Sun Yat-sen University has the greatest international influence. These institutions are significant not only in publication quantity but also in impact. Notably, the University of Texas System, despite ranking eighth in publication count (45 publications), holds the top spot in betweenness centrality (0.11 centrality), suggesting its research is highly collaborative internationally and highly influential in tumor immune escape.

**Table 2:** Top 15 core institutions in terms of publications.

Rank	Institution	NP	Centrality	Country
1	Sun Yat Sen University	72	0.09	China
2	Chinese Academy of Sciences	69	0.05	China
3	Central South University	55	0.04	China
4	Shanghai Jiao Tong University	55	0.02	China
5	Chinese Academy of Medical Sciences - Peking Union Medical College	54	0	China
6	Fudan University	48	0.02	China
7	Zhejiang University	46	0.09	China
8	University of Texas System	45	0.11	USA
9	Institut National de la Sante et de la Recherche Medicale (Inserm)	44	0.07	France
10	Huazhong University of Science & Technology	40	0.06	China
11	Helmholtz Association	38	0.05	Germany
12	Nanjing Medical University	36	0.01	China
13	University of California System	35	0.04	USA
14	Southern Medical University - China	34	0.01	China
15	Harvard University	33	0.01	USA

NP, number of publications.

**Figure 5:**
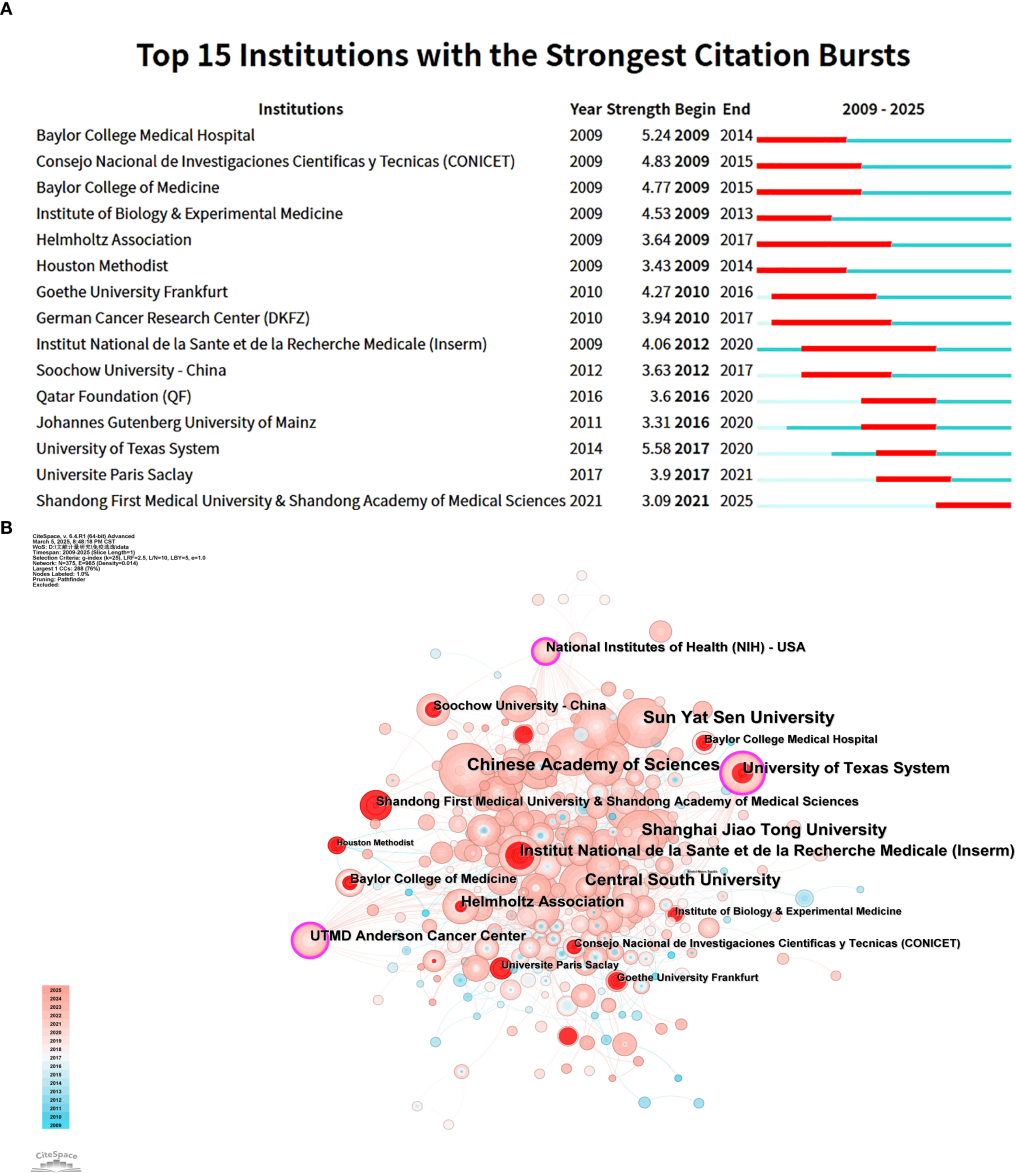
This analysis focuses on research institutes related to tumor immune escape. **(A)** It visualises co - author and research institution collaborations in this field. **(B)** It presents a co - occurrence mapping of research institutions. Here, node size shows co - occurrence frequency, and links indicate co - occurrence relationships. Nodes with purple circles have high betweenness centrality (≥0.1).

**Figure 5A** shows the top 15 institutions with citation outbreaks. Shandong First Medical University & Shandong Academy of Medical Sciences have recently experienced citation bursts, indicating significant potential in tumor immune escape research. In the co - occurrence graph (**Figure 5B**), node size represents co - occurrence frequency, and links show co - occurrence relationships. Nodes with purple rounded corners have high betweenness centrality (≥0.1), such as the University of Texas System and UTMD Anderson Cancer Center, which play key roles in connecting diverse research communities.

### 3.3 Analysis of journals

To identify active and influential journals in tumor immune escape, a visual analysis of published journals was done, uncovering 1,839 related publications in 472 academic journals. FRONTIERS IN IMMUNOLOGY had the most publications (126), followed by CANCERS (54) and CANCER RESEARCH (31) (see **Table 3**). Notably, CANCER RESEARCH has the highest impact factor (12.5) and average citations (107) among the top 10 journals, underscoring its significant impact in tumor immunology.

**Table 3:** Top 10 core journals.

Rank	Journal	h_index	NP	TC	AC	2024 JCI division	IF (2024)
1	FRONTIERS IN IMMUNOLOGY	36	126	4034	32	Q1	5.7
2	CANCERS	23	54	1508	27	Q2	4.5
3	CANCER RESEARCH	22	31	3321	107	Q1	12.5
4	ONCOIMMUNOLOGY	22	30	1484	49	Q1	6.5
5	FRONTIERS IN ONCOLOGY	19	54	1792	33	Q2	3.5
6	PLOS ONE	19	23	1893	82	Q1	2.9
7	CANCER IMMUNOLOGY RESEARCH	18	20	1267	63	Q1	8.9
8	JOURNAL FOR IMMUNOTHERAPY OF CANCER	18	33	1259	38	Q1	10.3
9	INTERNATIONAL JOURNAL OF MOLECULAR SCIENCES	16	34	781	22	Q2	4.9
10	JOURNAL OF IMMUNOLOGY	16	19	1981	104	Q2	3.6

h_index, Hirsch index; NP, number of publications; TC, total citation; AC, average citations; IF, impact factor.

**Figure 6:**
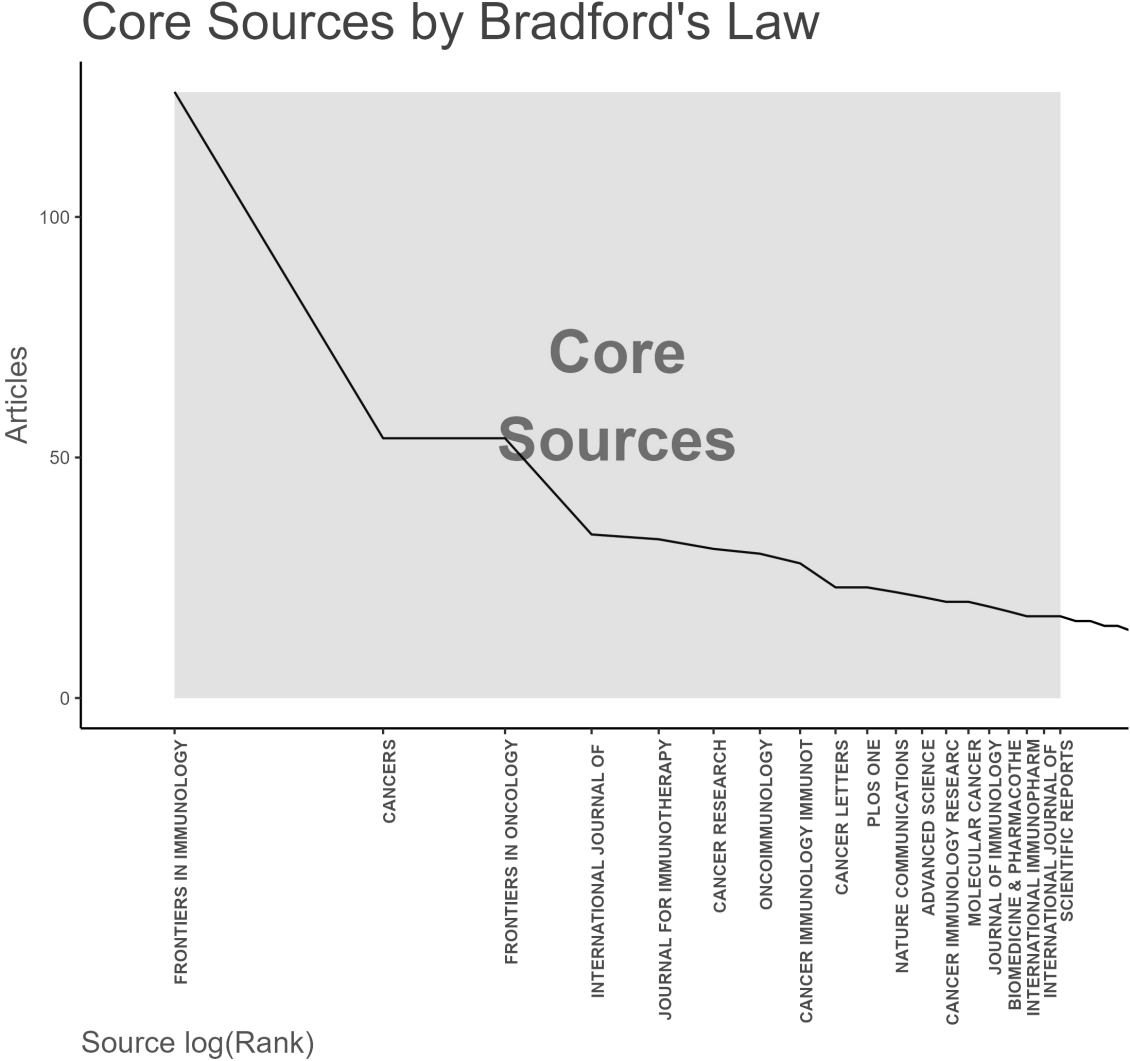
Analysis of academic journals related to tumor immune escape. Bradford’s law in academic journals.

**Figure 6** Application of Bradford’s Law showing core journals for tumor immune escape research.

**Figure 7:**
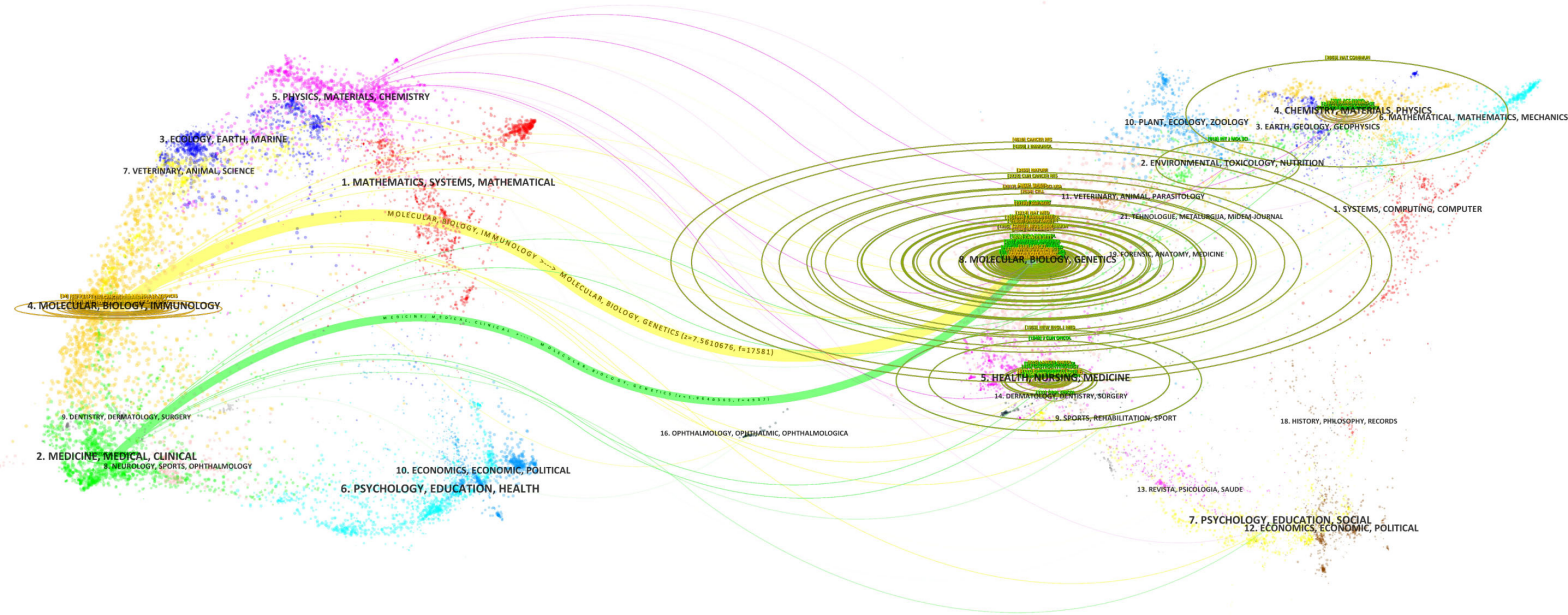
Double image overlay of journals in tumor immune escape research. This overlay visualises citation relationships between journals in this field. The lower graph shows citing journals on the left, cited journals on the right, with coloured lines indicating citation paths.

The double figure overlay reveals a single citation pathway in numerous inter - field links between journals (**Figure 7**). Interestingly, publications on MOLECULAR, BIOLOGY, GENETICS are mainly cited by publications on MOLECULAR, BIOLOGY, IMMUNOLOGY and MEDICINE, MEDICAL, CLINICAL.

### 3.4 Author contributions and co-occurrence

In this study, 12,322 authors were involved in the study. **Table 4** lists the top 10 most prominent authors in tumor immune escape research. Xuitao Cao leads the list with 11 articles and 1737 citations, followed by Kebin Liu with 10 articles and 558 citations. Notably, Li Yong has published fewer articles but has the second highest number of citations. His 2018 publication was the first article on tumor immune escape, marking him as a highly promising emerging figure in the field (**Table 4**).

**Table 4:** The ten most relevant authors and their works.

Rank	Author	h_index	NP	TC	PY_start
1	CAO XUETAO	9	11	1737	2009
2	LIU KEBIN	9	10	558	2016
3	RABINOVICH GABRIEL A.	9	11	590	2009
4	ELKORD EYAD	8	9	1049	2016
5	GUO WEI	8	10	680	2017
6	KOCH JOACHIM	8	8	403	2010
7	KOEHL ULRIKE	8	8	438	2010
8	LI WEI	8	9	342	2010
9	LI YONG	8	8	1609	2018
10	LU CHUNWAN	8	10	540	2016

NP, number of publications; TC, total citation; h_index, Hirsch index.

**Figure 8:**
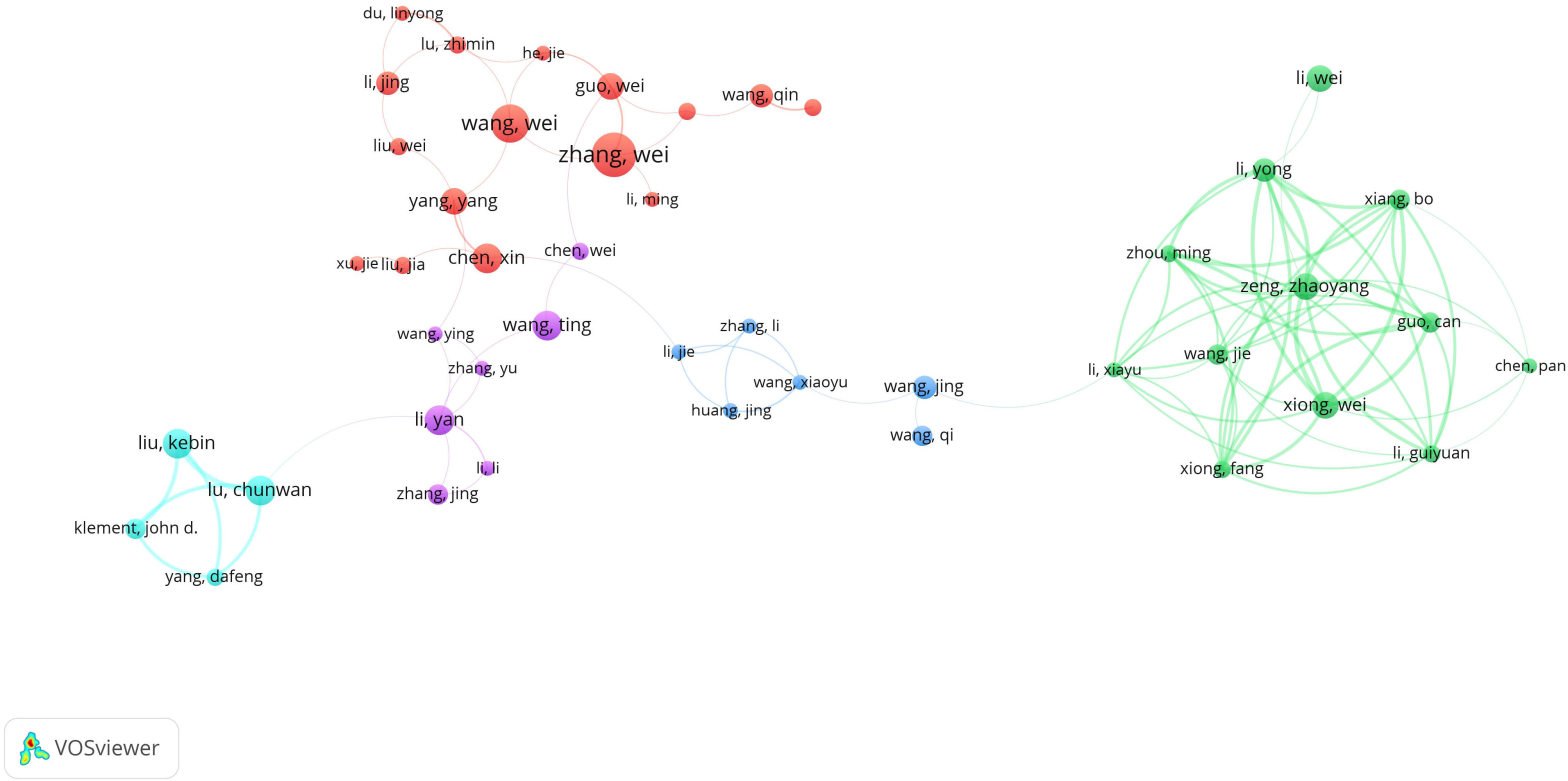
Authors’ tumor immune escape study blood infection co-occurrence graph. 744 different colored nodes reflect authors in different clusters. Node size indicates co-occurrence frequency and links indicate co-occurrence relationships between authors.

**Figure 8** illustrates the collaborative network of 45 authors who have published five or more articles. These authors are clustered into five distinct collaborative groups. While each group demonstrates strong internal collaboration, there is limited interaction between groups. This pattern indicates a relative lack of intergroup communication and suggests the need to strengthen inter-institutional and international collaboration within the field.

### 3.5 Citation and reference analyses

**Table 5** resents the top 10 most cited articles on tumor immune escape. The first two articles, each with over 2,800 citations, lead significantly over the remaining entries, underscoring their substantial influence in the field.

**Table 5:** Top 10 core literatures.

Rank	Title	First author	Journal	Type	Year	Total citations
1	Signatures of T cell dysfunction and exclusion predict cancer immunotherapy response	Jiang P	Nature Medicine	Article	2018	3151
2	PD-1 blockade with nivolumab in relapsed or refractory Hodgkin’s lymphoma	Ansell SM	New England Journal of Medicine	Article	2015	2834
3	Transforming Growth Factor-β Signaling in Immunity and Cancer	Batlle E	Immunity	Review	2019	1499
4	Targeting CXCL12 from FAP-expressing carcinoma-associated fibroblasts synergizes with anti–PD-L1 immunotherapy in pancreatic cancer	Feig C	Proceedings of The National Academy of Sciences of The United States of America	Article	2013	1481
5	LDHA-Associated Lactic Acid Production Blunts Tumor Immunosurveillance by T and NK Cells	Brand A	Cell Metabolism	Article	2016	1256
6	PD-1 and PD-L1 Checkpoint Signaling Inhibition for Cancer Immunotherapy: Mechanism, Combinations, and Clinical Outcome	Alsaab HO	Frontiers in Pharmacology	Review	2017	1237
7	Increased circulating myeloid-derived suppressor cells correlate with clinical cancer stage, metastatic tumor burden, and doxorubicin–cyclophosphamide chemotherapy	Diaz-Montero CM	Cancer Immunology, Immunotherapy	Article	2009	993
8	Role of the tumor microenvironment in PD-L1/PD-1-mediated tumor immune escape	Jiang XJ	Molecular Cancer	Review	2019	974
9	T-Cell Transfer Therapy Targeting Mutant KRAS in Cancer	Tran E	New England Journal of Medicine	Article	2016	966
10	Immune checkpoint blockade therapy for cancer: An overview of FDA-approved immune checkpoint inhibitors	Hargadon KM	International Immunopharmacology	Review	2018	872

The most cited article globally is ‘Signatures of T cell dysfunction and exclusion predict cancer immunotherapy response’ by Jiang P, published in Nature Medicine in 2018 with 3,151 citations. This article introduces TIDE, an alternative biomarker for predicting immune checkpoint blockade (ICB) response, offering novel ideas for immune checkpoint blockade prediction and laying the foundation for immunotherapy prognosis forecasting (20).

The second most cited article globally is Ansell SM’s ‘PD-1 blockade with nivolumab in relapsed or refractory Hodgkin’s lymphoma’, published in the New England Journal of Medicine in 2015. This study presents nivolumab as a new PD-1 blockade antibody and is the first to evaluate its efficacy and safety in relapsed or refractory Hodgkin’s lymphoma, providing a crucial basis for subsequent clinical applications in combating tumor immune escape (21).

**Figure 9:**
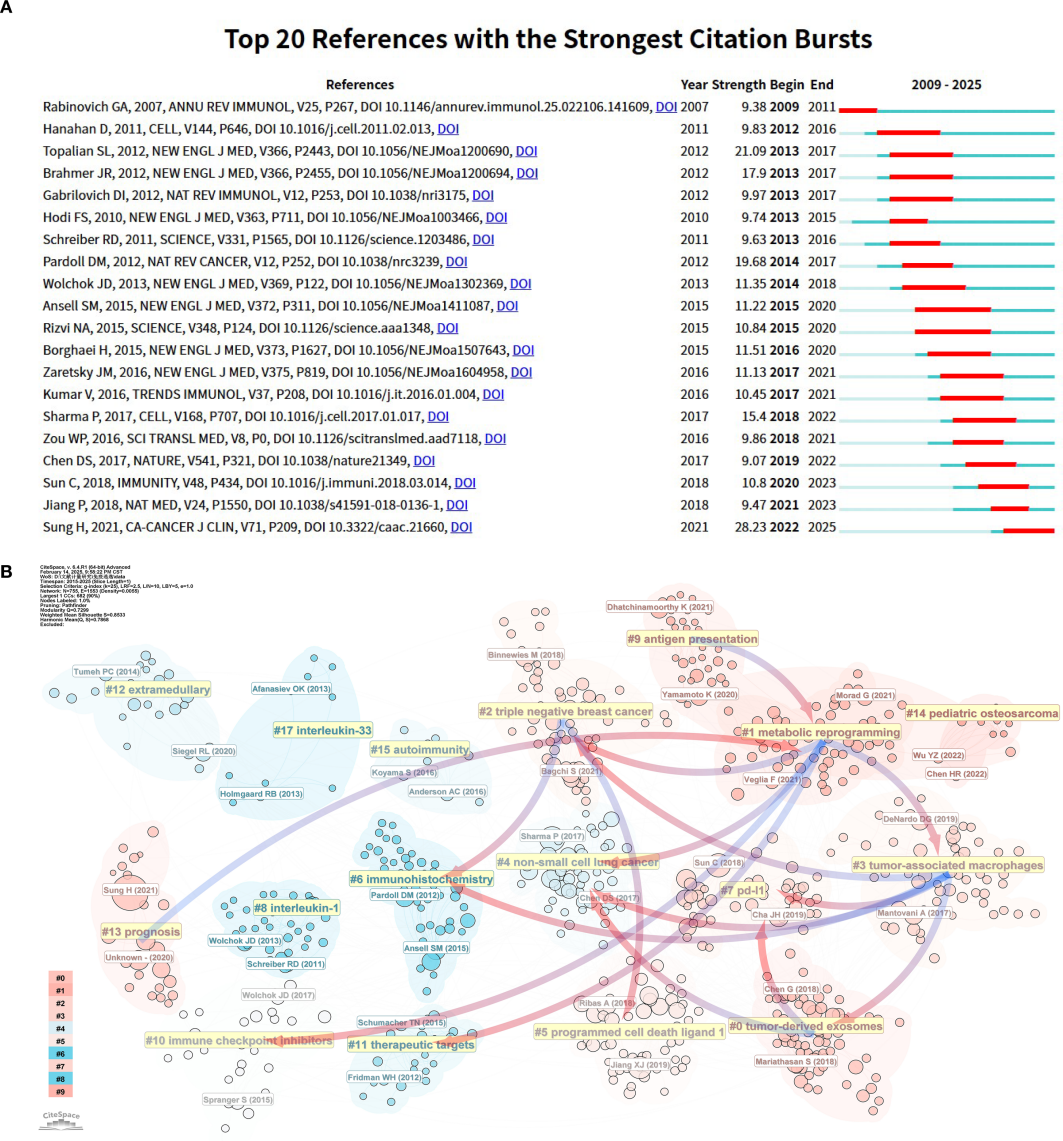
Analysis of references related to tumor immune escape. **(A)** The top 20 references with a significant increase in citation frequency. **(B)** Clustering of references according to similarity. Topics include #0 Tumorderived exosomes, #1 Metabolic reprogramming, #2 Triple-negative breast cancer, #3 Tumor-associated macrophages, #4 Non-small cell lung cancer, #5 PD-L1, #6 Immunohistochemistry, #7 PD-L1, #8 Interleukin-1 and so on. Linkage represents connections between different clusters, and the blue groups on the line evolve from the red ones.

**Figure 9A** highlights 20 core publications that experienced significant citation bursts, underscoring their influence and cutting-edge contributions to the tumor immune escape field during the analyzed timeframe. Early foundational literature, such as the work by Rabinovich et al. (22), experienced a dramatic citation surge between 2009 and 2011. This article became a landmark in tumor immunology by synthesizing previously fragmented immune escape mechanisms into a unified conceptual framework. It identified key immunotherapeutic targets, addressed unmet needs in immunotherapy research, and catalyzed the translation of basic science into clinical practice. Building upon earlier conceptual frameworks, Hanahan D provided a comprehensive synthesis of the hallmarks of cancer, in which immune evasion was recognized as an emerging hallmark and the TME was emphasized as a critical component influencing tumor progression and therapeutic resistance (23). This conceptual integration laid important theoretical groundwork for subsequent research into the mechanisms of tumor immune escape.

Between 2013 and 2017, three citation burst references were identified, all of which were pivotal clinical trials (24–26). This period marked a significant turning point, as immune checkpoint inhibitors (ICIs) transitioned from preclinical exploration to clinical application. Among these, the landmark study by Hodi FS et al. (26) demonstrated that ipilimumab significantly improved overall survival in patients with advanced melanoma (median OS increased from 6.4 to 10.1 months), leading to its FDA approval in 2011 for metastatic melanoma. Ipilimumab thereby became the first immune checkpoint inhibitor approved globally, inaugurating a new era in cancer immunotherapy. Four additional citation burst articles identified between 2015 and 2020 (21, 27–29) focused on PD-1 inhibitors, likely reflecting the momentum generated by the FDA approvals of nivolumab and pembrolizumab in 2014. These approvals marked a major breakthrough in immunotherapy and spurred a surge of clinical and translational research into immune checkpoint blockade strategies. In 2018, Jiang P and colleagues (20) developed TIDE (Tumor Immune Dysfunction and Exclusion), a computational framework designed to model the two major mechanisms of tumor immune escape and predict responses to ICI therapy. Beyond its direct predictive value, TIDE played a pioneering role in bridging AI and tumor immunology, setting the stage for subsequent applications of machine learning in decoding immune evasion. Notably, this study attracted widespread attention between 2021 and 2023 and remains the most cited article in the field to date, underscoring its foundational significance and groundbreaking impact. The article exhibiting the most intense citation burst was “Global Cancer Statistics 2020: GLOBOCAN Estimates of Incidence and Mortality Worldwide for 36 Cancers in 185 Countries” by Sung H et al. (30), published in 2021 in CA: A Cancer Journal for Clinicians (impact factor: 503.1). Utilizing GLOBOCAN 2020 data, this study provided a comprehensive overview of the global cancer burden, highlighting substantial regional differences in cancer incidence and mortality, and exploring the underlying epidemiological factors. The publication has since served as both an authoritative data source and an essential reference for global oncology research and clinical practice.

The use of co - citation cluster analysis offers an objective illustration of the knowledge structure within a research area. For a more detailed description of the co - cited reference groups, a network diagram was generated. The degree of association between articles was categorized into 17 groups, which formed the basis for the clustering classification. The co - citation cluster analysis, as shown in the diagram, clearly reveals the knowledge structure of the research area. To fully describe the co - cited literature groups, a complex network diagram was constructed (as shown in **Figure 9B**). Research topics were classified into 17 categories based on co - citation relationships, forming a clear cluster structure. In this diagram, (1) metabolic reprogramming, being the largest cluster, indicates that metabolic reprogramming - related research holds a central position in the field and carries extensive academic influence.

Evidence of evolution over time among the study clusters is also apparent. For instance, the gradual evolution of (4) non-small cell lung cancer clusters into the emergence of (0) tumor-derived exosome, (3) tumor-associated macrophages and (1) metabolic reprogramming clusters. In the field of non-small cell lung cancer research, the transition from conventional to immunotherapeutic approaches has catalyzed the rapid evolution of tumor immunotherapy. Additionally, triple negative breast cancer is the most aggressive breast cancer type, having limited treatment choices and a poor prognosis (31). The figure shows that the triple negative breast cancer group gradually evolved into metabolic reprogramming and tumor-associated macrophage groups. This means immunotherapy breakthroughs bring hope to triple negative breast cancer clinical treatment.

The (7) PD-L1 cohort has gradually evolved into the (3) tumor-associated macrophages and (0) tumor-derived exosomes cohorts, reflecting the research process of tumor immune escape, which further promotes the exploration of the tumor microenvironment due to the differentiated efficacy of PD-L1-targeted drugs. It reflects the in-depth exploration of the mechanism of tumor development and the continuous optimization of immunotherapy strategies in the field of tumor immunity.

Beyond the main evolutionary trends, several smaller groups indicate the ongoing development of specific research directions. Notably, (1) metabolic reprogramming cohorts have gradually evolved into (9) prognosis cohorts. This shift shows that as tumor metabolomics advances, researchers are paying more attention to predicting patient prognosis. Metabolic reprogramming is a key strategy for tumor cells to adapt to harsh microenvironments and maintain rapid proliferation and survival (32, 33). This metabolic alteration not only supports tumor growth but also closely correlates with tumor malignancy and poor patient prognosis (34), providing a solid theoretical basis for the evolution of metabolic reprogramming research towards prognosis. In clinical practice, accurately predicting patient prognosis is crucial for developing personalized treatment plans and improving survival rates.

### 3.6 Keywords co-occurrence analysis

**Figure 10:**
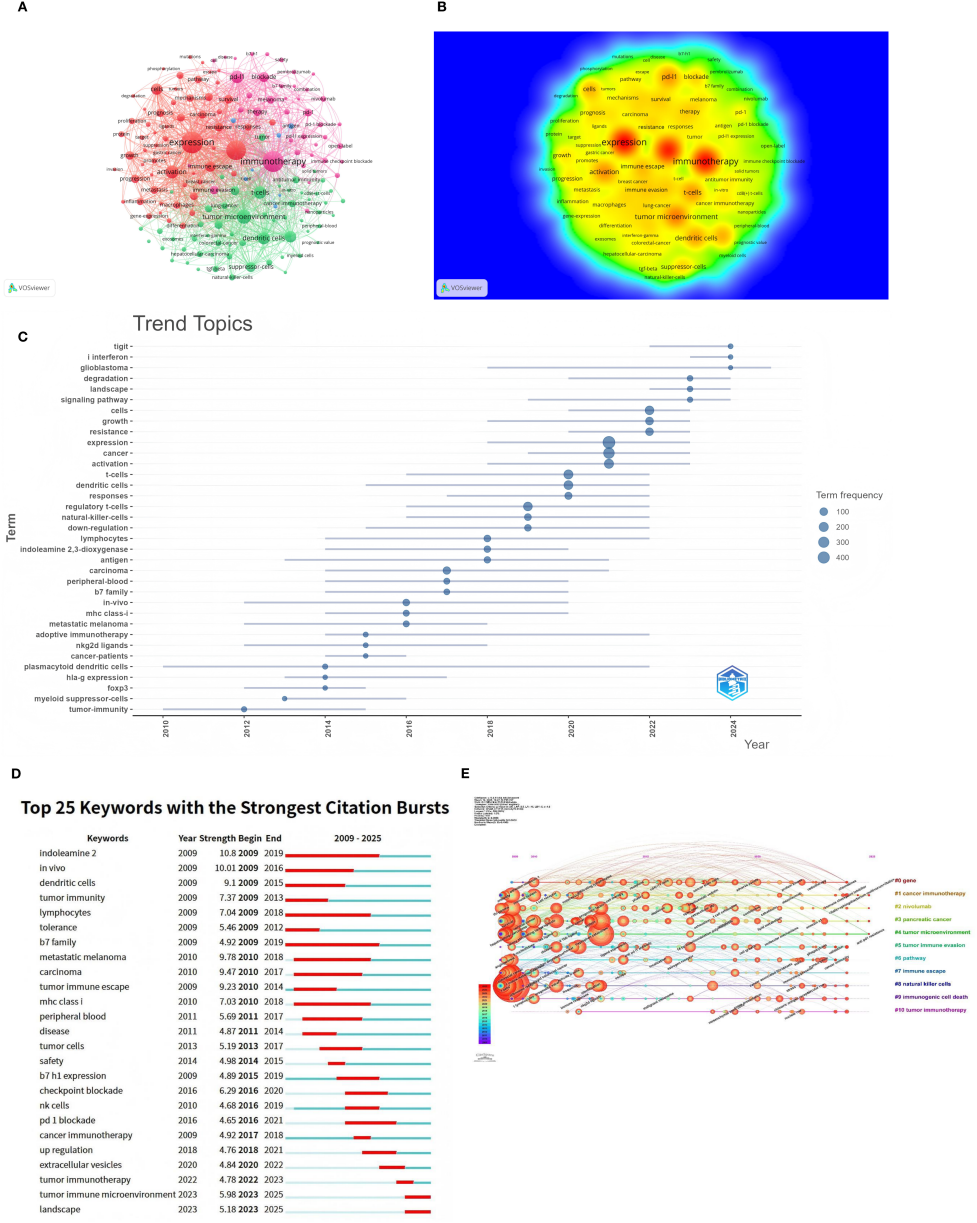
Keyword co-occurrence analysis in tumor immune escape studies. **(A)** Network visualisation for keyword co-occurrence analysis (n>5). Each node in the network represents a keyword, with the size of the node indicating the number of times the keyword occurs. Lines between nodes indicate co-occurrence between keywords; the larger the node size, the higher the frequency of the keyword. **(B)** Density visualisation of keyword co-occurrence analysis. This visualisation methodically illustrates the density and intensity of research themes within the designated field. Heat maps are utilised to accentuate areas of varying research intensity, with warmer colours denoting higher activity and stronger connections. **(C)** Trend themes from 2009 to 2025. The timeline illustrates the temporal progression of pivotal research themes within the field. The relative prominence of these research themes undergoes substantial fluctuations over the course of time, with larger nodes denoting elevated frequency and significance. **(D)** The 25 keywords with the strongest citation bursts are displayed. The blue line indicates the time axis, with the red segments denoting the start year, end year, and duration of each burst. **(E)** Timeline of keyword co-occurrence analysis. The timeline visualises the temporal evolution of key research topics in the field. The salience of each keyword undergoes a change over time, with larger and more concentrated nodes representing higher frequency and importance. The keywords are then organised into clusters on the right-hand side of the figure.

**Figures 10A, B** highlight key themes in tumor immune escape research, including ‘immunotherapy’, ‘pd-l1’, ‘cancer’, ‘expression’, ‘tumor microenvironment’, ‘dendritic cells’, and ‘t-cells’. These keywords are strongly interconnected, reflecting their central role in the research community. The density visualization reveals intense research activity around these themes, with warmer colors indicating areas of high interest.

**Figures 10C, D** depict the evolution of research topics in tumor immune evasion. **Figure 10C** analysis of theme word trends from 2009 to 2025 shows that “TIGIT,” “type I interferon,” and “glioblastoma” are the next few years’ research Frontiers. Keyword burst analysis delineates three pivotal evolutionary stages in tumor immune escape research (**Figure 10D**). The pronounced bursts of “dendritic cells” and “lymphocytes” during 2009–2015 underscored foundational investigations into antigen presentation machinery and T-cell activation dynamics. Concurrently, sustained bursts of “indoleamine 2,3-dioxygenase” and “B7 family”(2009–2019) signaled the emergence of metabolic checkpoint pathways as critical regulators. The subsequent phase (2016–2021) witnessed transformative clinical advances, where bursts in “checkpoint blockade” and “PD-1 blockade correlated with therapeutic breakthroughs in immune checkpoint inhibitors, while renewed focus on “B7-H1 expression” (2015-2019) reflected its consolidation as a predictive biomarker. Current research has shifted toward tumor microenvironmental orchestration, exemplified by the burst in extracellular vesicles (2020–2022) highlighting exosome-mediated immune remodeling. Dominant ongoing bursts for tumor immune microenvironment and landscape (2023–2025) reveal accelerating adoption of spatial multi-omics and integrative biology frameworks to deconvolute immune evasion ecosystems. Notably, persistent attention to MHC class I (2010–2018) reflects enduring challenges in antigen presentation defects as core resistance mechanisms. **Figure 10E** categorizes keywords into 11 groups arranged chronologically, showing the most recent and prevalent keywords as ‘Immune Checkpoint Inhibitors’, ‘Sphingobacterium multivorum’, and ‘Anti PD-1 Resistance’.

## 4 Discussion

### 4.1 Overall distribution

Research on tumor immune evasion has sustained growth from 2009 to 2024, with no sign of abating in 2024. This reflects growing interest and attention in the field (30). Immunotherapy has become a major strategy for cancer treatment. However, tumor immune escape remains a significant challenge to the efficacy of anticancer therapies. To understand the mechanism of tumor immune escape, many targeted approaches have been explored, and some drugs have been clinically applied and achieved better efficacy (35, 36). Globally, China, the United States, and European countries are major contributors to research on tumor immune evasion. These countries’ research efforts have a clear advantage in addressing the global cancer threat. China leads in research output with 958 (52.1%) research papers published, showing the breadth and depth of its research and its great global influence. This is closely related to China’s comprehensive cancer screening and registration system and the rising cancer mortality rate (37, 38). Meanwhile, the USA and Germany have published 281(15.3%) and 114(6.2%) publications respectively, showing that the USA and Europe also have a large influence in the field of tumor immune escape. China’s cancer screening program, initiated in 1958, has significantly expanded its coverage over the past decade. This provides robust and credible primary data supporting research in the field of tumor immune escape (38). Presently, China faces a substantial cancer burden. According to the latest World Health Organization (WHO) estimates, the country accounts for 24% of global cancer incidence cases, with cancer mortality rates exceeding the global average (1, 39, 40). Consequently, the “Healthy China 2030” initiative designates cancer prevention and control as a strategic priority. Coupled with sustained investment in scientific research, these policies have enabled China to achieve significant progress in cancer prevention and treatment, while making substantial contributions to tumor immune escape research (41).

In the field of research institutions, China’s top institutions excel in output and influence. All seven institutions with the highest number of relevant publications are in China. Sun Yat-sen University leads with 72 publications and a betweenness centrality of 0.09, showing its significant position and global collaborative contribution in tumor immune evasion research. Although the University of Texas System has fewer publications, its betweenness centrality of 0.11 reflects substantial contributions to global cooperation in this field. Notably, Shandong First Medical University & Shandong Academy of Medical Sciences has rapidly risen to become a prominent player in recent years. Despite strong domestic collaborations among Chinese institutions, international cooperation remains relatively limited, which may hinder overall progress in tumor immune evasion research. Therefore, strengthening international collaborations between research institutions is crucial for accelerating global research efforts and addressing the worldwide cancer challenge.

Bibliometric analysis shows the USA and France excel in international collaboration, especially in cross - national publications. The UK, despite fewer publications, maintains strong research networks with other countries, highlighting its role in advancing global tumor immune escape research. The United States not only demonstrates strong research output in the field of tumor immune escape research, but also maintains a high proportion of multinational collaborative publications. That edge traces back to decades of steady investment by the National Cancer Institute in fostering worldwide oncology partnerships (42). Additionally, the Cancer Genome Atlas (TCGA) (43) have established accessible genomic databases that have catalyzed the formation of multinational research consortia and promoted collaborative discoveries. These collaborations facilitate knowledge sharing and enhance international synergies, enabling effective responses to global cancer challenges. Notably, low - and middle - income countries, largely due to China’s contributions, have significantly contributed to this research, helping overcome resource deficiencies and high cancer risks in these areas (44). Nevertheless, cross - border collaboration between research institutions remains limited, which may impede research progress. Enhanced global research collaboration and resource sharing could facilitate tumor immune escape research in these regions and provide more diverse perspectives and data support in the global fight against cancer.

Among journals, Frontiers in Immunology publishes the most tumor immune escape studies, far exceeding other academic journals. While Cancer Research publishes fewer articles, it has the highest average citations and impact factor (12.5), underscoring its substantial influence in this field. Among authors, Cao XT from China is the most prolific and cited. He and his team summarize the role of tumor - associated macrophages (TAMs) in promoting tumor progression and drug resistance (45), explore antibody variable region engineering applications, and discuss future antibody engineering directions to enhance cancer therapy (46).

### 4.2 Evolution of research focus and translational impact

Our bibliometric analysis shows that tumor immune escape research has evolved from focusing on classical checkpoints like PD-1/PD-L1 to exploring more complex mechanisms such as T cell exclusion, antigen presentation loss, and TME dynamics. (**Table 6**) Recently, emerging hotspots include combination immunotherapies, AI-assisted predictive modeling, and the cross-talk between metabolic reprogramming and the microbiome—an area gaining notable traction.

**Table 6:** Major molecular and cellular drivers of tumor immune evasion.

Category	Molecule/Cell	Definition	Mechanism of action
Immune Checkpoints	PD-L1	Transmembrane immunosuppressive protein	Binds PD-1 on T cells to inhibit activation and cytokine production
	CTLA-4	T-cell surface receptor	Competes with CD28 for B7 ligands on APCs, blocking co-stimulatory signals
Antigen Presentation	HLA-I	Major histocompatibility complex class I	Presents tumor antigens to CD8+ T cells; frequent allelic loss in tumors
	B2M	β2-microglobulin subunit	Essential for MHC-I complex stability; mutations cause antigen presentation failure
	TAP1/2	Transporter associated with antigen processing	Transports antigen peptides to ER for MHC-I loading; often downregulated in tumors
Suppressive Cytokines	TGF-β	Pleiotropic immunosuppressive cytokine	Induces Treg differentiation; blocks CD8+ T-cell proliferation; disrupts ribosomal P-stalk formation
	IL-10	Anti-inflammatory cytokine	Inhibits dendritic cell maturation; promotes M2 macrophage polarization; downregulates TAP1
Metabolic Regulators	IDO1	Tryptophan-catabolizing enzyme	Depletes tryptophan to induce T-cell anergy; generates kynurenine to activate Tregs
	CD73	Ecto-5’-nucleotidase	Converts AMP to adenosine, which binds A2AR on T cells to suppress activation
TME Suppressive Cells	Tregs	Regulatory T cells (CD4+CD25+FoxP3+)	Express CTLA-4 to deplete CD80/86 on DCs; secrete IL-10/TGF-β; directly kill CD8+ T cells via granzyme B
	M2 Macrophages	Alternatively activated macrophages	Secrete arginase-1 to deplete arginine (essential for T cells); produce VEGF for angiogenesis; express PD-L1
	CAFs	Cancer-associated fibroblasts	Secrete CXCL12 to block T-cell infiltration; produce TGF-β to induce T-cell exhaustion; create physical barriers

These trends have clear translational value. Insights into immune evasion are driving the development of multi-target therapeutic strategies, particularly for tumors resistant to standard immunotherapies, such as glioblastoma (GBM) and MSS colorectal cancer. Advances in single-cell profiling, spatial transcriptomics, and AI tools are further enabling precision immune phenotyping and personalized treatment planning.

At the policy level, the increasing relevance of immune escape calls for integrating immune profiling into clinical workflows and national treatment guidelines. Promoting international collaboration and investment in emerging technologies will be key to accelerating progress and improving global cancer outcomes.

### 4.3 Research hotspots

Bibliometrics is crucial for processing and analyzing large-scale data to offer researchers insights into trends. Analyzing frequent keywords and subject terms can uncover changing trends and key themes, which are vital for understanding the field’s evolution. Based on the above analysis, current major hotspots focus on areas like Immune Checkpoint Inhibitors, tumor immune microenvironment, and landscape. An in-depth analysis of these research hotspots can help better understand the progress of tumor immune escape research and predict future developments in the context of current studies.

#### 4.3.1 Current research hotspots

##### 4.3.1.1 Novel immune checkpoint discovery and multi-target combination strategies

Antibodies targeting immune checkpoints such as the programmed death-1 (PD-1) receptor, its ligand PD-L1, or cytotoxic T lymphocyte-associated-4 (CTLA-4) have transformed the treatment of many tumor types. However, only a small percentage of patients produce a durable response. Consequently, researchers are actively exploring new immune checkpoints to target and combining therapies to achieve enhanced therapeutic efficacy.

TIGIT, a member of the poliovirus receptor (PVR)/nectin family, is expressed on T cells, NK cells, and Tregs (47, 48). It features an extracellular IgV domain, transmembrane region, and cytoplasmic ITIM/ITT motifs. By binding to CD155 (PVR) with high affinity, TIGIT competitively inhibits the co-stimulatory receptor DNAM-1 (CD226), thus suppressing the activation of T and NK cells (49, 50). The signaling pathway involved in this process, mediated by Grb2/SHIP1, results in the disruption of the MAPK and NF-κB pathways. In clinical trials, the anti-TIGIT antibody tiragolumab, when combined with the anti-PD-L1 antibody atezolizumab, demonstrated an improved overall response rate (ORR) of 37.3%, compared to 20.6% with monotherapy, in patients with PD-L1-high non-small cell lung cancer (NSCLC) (51).

LAG-3 (Lymphocyte Activation Gene 3), a member of the immunoglobulin (Ig) superfamily located on chromosome 12, is expressed on CD4+/CD8+ T cells, natural killer (NK) cells, regulatory T cells (Tregs), and plasmacytoid dendritic cells (52, 53). It inhibits T cell activation by binding with high affinity to MHC-II molecules on antigen-presenting or tumor cells, suppressing IL-2 and IFN-γ secretion. The intracellular domain of LAG-3 contains the S484, KIEELE, and EP motifs, which regulate cellular localization and TCR signaling (54, 55). In clinical trials, relatlimab (anti-LAG-3 monoclonal antibody) combined with nivolumab (anti-PD-1) achieved FDA approval for advanced melanoma (Phase III RELATIVITY-047 trial), demonstrating superior progression-free survival (PFS: 10.1 *vs*. 4.6 months) (56). Future multi-targeted combination therapies have great potential in the treatment of cancer.

##### 4.3.1.2 NETs–TME interactions: a hotspot in tumor immunology

The tumor microenvironment (TME) is now regarded as crucial in cancer development, progression, and treatment (57). This heterogeneous system consists of a chemical TME (marked by acidic pH, hypoxia, and low nutrition), a cellular TME (including tumor cells, stromal cells, pericytes, endothelial cells, immune cells, and the extracellular matrix), and various signaling molecules like cytokines, chemokines, and growth factors within the microenvironment (58, 59). These components interact closely and consistently with tumor cells, thereby enabling the tumor to evade the immune system through different mechanisms (60).

Among the immune cells within the TME, neutrophils play a central role in all stages of cancer progression (61). Neutrophils contribute to tumor metastasis through the formation of extracellular traps (NETs), which protect tumors from effector T cell-mediated elimination (62). However, NETs may also have a dual role in the TME. In certain acute inflammatory conditions, NETs have been shown to inhibit melanoma cell migration and promote tumor lysis, suggesting that they can contribute to tumor elimination (63). Interestingly, depletion of the immune checkpoint receptor CD276 has been found to significantly reduce the expression of CXCL1, which ultimately diminishes neutrophil infiltration into tumors, thereby decreasing NET formation through the CXCL1-CXCR2 axis. This reduction in neutrophil-driven immune suppression can enhance NK cell infiltration, which may play a pivotal role in halting the progression of esophageal squamous cell carcinoma (64). Chronic stress has been shown to disrupt the normal circadian rhythm of neutrophils, leading to increased formation of neutrophil extracellular traps (NETs) via elevated glucocorticoid release. This alteration progressively shapes a TME that favors metastatic cancer progression (65).

Understanding the TME and the role of neutrophil extracellular traps (NETs) is essential for advancing cancer immunology. The TME serves not only as a physical and biochemical scaffold for tumor growth but also as a dynamic immunological hub that orchestrates immune evasion, metastatic potential, and therapeutic resistance. Moving forward, deeper investigation into the mechanistic crosstalk between NETs and the TME is critical. Unraveling these interactions will inform the development of novel immunotherapeutic strategies, including NET-targeted interventions, which may reshape the immunosuppressive landscape of solid tumors and improve clinical outcomes.

##### 4.3.1.3 Type I interferon as a promising strategy to overcome ICB resistance

Despite the transformative success of immune checkpoint blockade therapies, their clinical efficacy remains highly heterogeneous across patient populations (66). A considerable proportion of patients exhibit primary resistance or develop adaptive resistance during treatment, often due to a highly immunosuppressive TME, insufficient tumor immunogenicity, or impaired effector immune responses (67). Type I interferons (IFN-I) have emerged as key modulators of antitumor immunity. IFN-I signaling coordinates a range of immune-regulatory processes, including dendritic cell (DC) maturation, CD8^+^ T cell activation, macrophage polarization, and the induction of tumor cell senescence and apoptosis (68). Notably, IFN-I can also act directly on natural killer T (NKT) cells, enhancing their infiltration into the TME and further amplifying the immune response (69). A promising approach to overcoming ICB resistance involves combining checkpoint inhibitors with IFN-I–activating strategies, particularly in tumors characterized as immunologically “cold.” (70).

Borui Tang et al. identified daurisoline (DS)—a bioactive alkaloid extracted from the rhizomes of the traditional Chinese medicinal herb Ban Yue Zi—as a potent inducer of IFN-I signaling. Mechanistically, DS stimulates IFN-I production via a TANK–TBK1–dependent pathway in tumor cells. The IFN-I released subsequently promotes NKT cell recruitment, enhancing antitumor immune activity (71). Importantly, their study demonstrated that combination therapy using DS with either anti–PD-1 antibodies or the STING agonist diABZI significantly remodeled the immune landscape of the TME. These findings suggest that DS-based combinations may serve as a viable strategy to overcome resistance in ICB-refractory tumors.

Similarly, Ruixuan Liu et al. engineered a bacterial strain, VNP-C-C, that co-expresses CCL2 and CXCL9, thereby facilitating immune cell mobilization and establishing a pro-inflammatory TME. This strategy induces immunogenic cell death (ICD) and activates the cGAS–STING pathway, resulting in elevated IFN-I production and a strengthened antitumor response (72). Interestingly, following VNP-C-C treatment, a marked upregulation of PD-1 expression on tumor-infiltrating T cells was observed—indicative of robust immune activation but also potential T cell exhaustion and immune escape. These findings highlight VNP-C-C as a potential priming agent for ICB-based combination immunotherapy.

In summary, these preclinical studies underscore the emerging role of type I interferon signaling as a central modulator of resistance to immune checkpoint therapies. While both DS and VNP-C-C have shown promising immunomodulatory effects in experimental models, neither has yet advanced to clinical trials. Nevertheless, the ability of IFN-I–targeted interventions to convert “cold” tumors into “hot” ones positions this axis as a compelling focus for future translational research and therapeutic development.

#### 4.3.2 Future research hotspots

##### 4.3.2.1 Cross-cutting studies of metabolic reprogramming and microbiome

The intersection of the microbiome and metabolic reprogramming has emerged as a prominent research focus in the field of tumor immune evasion, particularly in the context of colorectal cancer. The microbiome plays a pivotal role in shaping the TME by modulating inflammation and immune responses (73, 74). It influences cancer initiation, progression, metastasis, and immune escape mechanisms through both microbial actions and their metabolites (75, 76). Microbial metabolites such as short-chain fatty acids (SCFAs) and bile acids reprogram metabolic processes within the TME, either enhancing or inhibiting immune responses. For example, SCFAs, particularly butyrate, produced by beneficial bacteria like Bacteroides thetaiotaomicron, help maintain immune balance by promoting regulatory T cell (Treg) differentiation, which can suppress inflammation and support immune homeostasis (77). In contrast, dysbiosis—often linked to poor dietary habits—can favor pathogenic bacteria like Fusobacterium nucleatum, which promotes immune evasion by inducing M2 macrophage polarization, suppressing T cell responses, and enhancing inflammation, ultimately accelerating cancer progression (78). Furthermore, the metabolic competition within the TME between cancer cells, immune cells, and microbes adds another layer of complexity to immune evasion. Tumor cells reprogram their metabolism to favor glycolysis, thus depriving immune cells of essential nutrients like glucose and glutamine. This metabolic shift impairs T cell function and promotes immune suppression, facilitating tumor progression (75). The crosstalk between tumor metabolism, microbial metabolites, and immune responses underscores the potential for targeting these metabolic pathways to improve the efficacy of immunotherapies (79). In conclusion, the intersection of metabolic reprogramming and the microbiome offers a promising avenue for cancer research, particularly in tumor immune evasion. By understanding how microbial metabolites influence tumor metabolism and immune responses, this area could lead to new therapies that enhance immunotherapy effectiveness. Further exploration of this cross-cutting research will be key to developing personalized treatments that combine microbiome and metabolic strategies to overcome immune suppression.

##### 4.3.2.2 Intelligent decoding of the tumor immune evasion landscape

As tumor immunology advances toward increasingly personalized and dynamic paradigms, the concept of an “immune evasion landscape” has emerged as a critical framework to describe the intricate, multidimensional interplay between tumors and the host immune system. Recent progress in high-throughput technologies—such as spatial transcriptomics (80), single-cell technologies (81, 82), and multi-omics integration (83)—has enabled unprecedented resolution in mapping this landscape. AI, empowered by access to high-dimensional biological datasets and breakthroughs in computational power and deep learning architectures, offers a transformative approach to decoding these complex interactions (84, 85). Several recent studies exemplify this trend. For instance, Hanqi Li et al. (86) integrated four histological dimensions to define three molecular subtypes of hepatocellular carcinoma (HCC), establishing an MSRS model validated through single-cell RNA sequencing, spatial transcriptomics, and functional assays. This model demonstrated robust prognostic capability and potential for guiding individualized therapy. In another study (87), researchers applied imaging mass cytometry and a graph-based AI model to compare non-small cell lung TMEs in people with and without HIV. Leveraging PageRank and diffusion maps, the model achieved 84.6% accuracy in classifying HIV-associated tumors and identified key immunosuppressive markers, such as PD-L2 on tumor-associated macrophages and CD25 on infiltrating T cells. Additionally, Liu et al. (88) developed a self-supervised learning (SSL) framework based on the Barlow Twins method to analyze over 1,600 H&E-stained colon cancer slides from TCGA-COAD and AVANT cohorts. Their model, trained without manual annotations, extracted latent features to define 47 histomorphological phenotype clusters (HPCs) that reflect immune infiltration, stromal disorganization, and tumor necrosis. The HPCs proved predictive of survival outcomes and treatment response, demonstrating how SSL can be leveraged for label-free, interpretable profiling of the TME. In summary, these advances highlight the growing synergy between AI and tumor immunology. By enabling mechanistic, data-driven characterization of the immune evasion landscape, AI models are not only enhancing prognostic precision but also uncovering biologically meaningful therapeutic targets (89). Looking ahead, the integration of multi-modal data—including spatial, transcriptomic, proteomic, and morphological inputs—into unified AI frameworks will likely revolutionize our ability to anticipate tumor immune dynamics and design next-generation precision immunotherapies tailored to individual patients.

##### 4.3.2.3 The future of glioblastoma: combination immunotherapy

GBM is the most common and aggressive form of primary brain tumor, characterized by a complex network of survival mechanisms that promote therapeutic resistance and immune evasion (90). Within its highly immunosuppressive TME, GBM stem cells—notorious for their intrinsic drug resistance—remain key contributors to treatment failure and disease recurrence (91). The blood–brain barrier further restricts the delivery of therapeutics, while tumor antigenic heterogeneity, limited neoantigen presentation, and T cell exclusion add layers of immune resistance (92). Thus, the most urgent challenge lies in designing integrated therapeutic strategies that concurrently target multiple immune escape mechanisms and reinforce the overall antitumor immune response (93).

The team led by Arrieta VA (94) used low-intensity pulsed ultrasound (LIPU) and intravenous microbubbles to open the blood-brain barrier and increase the concentration of liposomal doxorubicin and PD-1 blocking antibody. Additionally, it was found that when administered with LIPU/MB, doxorubicin’s efficacy surpassed simple drug delivery; it significantly modulated the TME, potentially improving the presentation of tumor antigens to T cells, thereby enhancing the efficacy of T cell-based immunotherapy (including PD-1 blockade).

Luo F et al. (95) found that LRRC15 expression was elevated in GBM patients who did not respond to anti-PD-1 therapy. Therefore, they believe that targeting LRRC15 may provide a new strategy to enhance anti-PD-1 therapy and overcome immune therapy resistance in GBM.

In the preclinical model developed by the Xing YL team (96), it was found that BRAFi+MEKi can synergize with ICI by enhancing T cell activity and antigen presentation, thereby increasing the intrinsic sensitivity of tumors. However, the combination therapy has significant toxicity. Therefore, they propose incorporating galectin-3 inhibitors into treatment regimens for these gliomas as a promising strategy to improve treatment efficacy while controlling toxicity, thereby enhancing patients’ overall quality of life.

In summary, while GBM remains highly resistant to current therapies, progress in blood–brain barrier-penetrating delivery systems, TME modulation, and biomarker-driven combinations has opened new avenues for immunotherapy. Future research should prioritize dissecting the immune evasion mechanisms—particularly those involving GBM stem cells, myeloid cells, and stromal factors—while advancing precision delivery technologies to enhance treatment efficacy. These efforts will be key to developing the next GBM of effective, personalized immunotherapeutic strategies for glioblastoma.

### 4.4 Limitations

This bibliometric analysis provides a comprehensive overview of tumor immune escape research, though several methodological limitations warrant acknowledgment. First, the keyword strategy, while designed for thematic specificity, may have inadvertently excluded conceptually related topics, leading to potential omissions in the broader immuno-oncology landscape. Second, exclusive reliance on the Web of Science Core Collection—though beneficial for standardization—may underrepresent applied or interdisciplinary studies more extensively indexed in databases such as PubMed or Scopus. Third, citation-based metrics are inherently time-sensitive, often disadvantaging recent publications and reflecting academic rather than translational impact. Fourth, the exclusion of non-English publications to ensure language consistency may introduce geographic bias, potentially overlooking contributions from non-English-speaking countries. Lastly, a certain degree of subjectivity is unavoidable in the interpretation and synthesis of bibliometric findings.

## 5 Conclusion and future perspectives

This bibliometric study provides a comprehensive overview of the intellectual landscape, research hotspots, and developmental trajectory of tumor immune escape research over the past 14 years. By mapping influential nation, authors, core journals, reference, and keyword bursts, this work not only summarizes major contributions in the field but also helps researchers better understand its evolution and emerging directions. Based on the observed patterns, we propose three key areas that warrant further exploration: (1) advancing interdisciplinary research at the intersection of the microbiome, metabolism, and immune regulation; (2) integrating artificial intelligence and multi-omics data to enhance predictive modeling and therapeutic precision; and (3) combining multi-modal therapeutic strategies to overcome immune escape more effectively.

Looking ahead, future research should emphasize translating mechanistic discoveries into clinically actionable strategies, particularly in identifying biomarkers that predict immune evasion and therapy resistance. Greater investment in large-scale, real-world immunotherapy data, along with the development of open-access, cross-platform analytical tools, will further support reproducibility and innovation. Moreover, fostering stronger international collaboration among researchers, institutions, and countries will be vital to accelerating discovery in this field and promoting the global advancement of cancer immunotherapy.

## Data Availability

The original contributions presented in the study are included in the article/**Supplementary Material**. Further inquiries can be directed to the corresponding author.
