# Supplementary figures and images for: Trends and hotspots in research related to tumor immune escape: bibliometric analysis and future perspectives
Source: Front Immunol. 2025 Aug 28;16:1604216. doi: 10.3389/fimmu.2025.1604216 (PMC12423061; doi:10.3389/fimmu.2025.1604216)

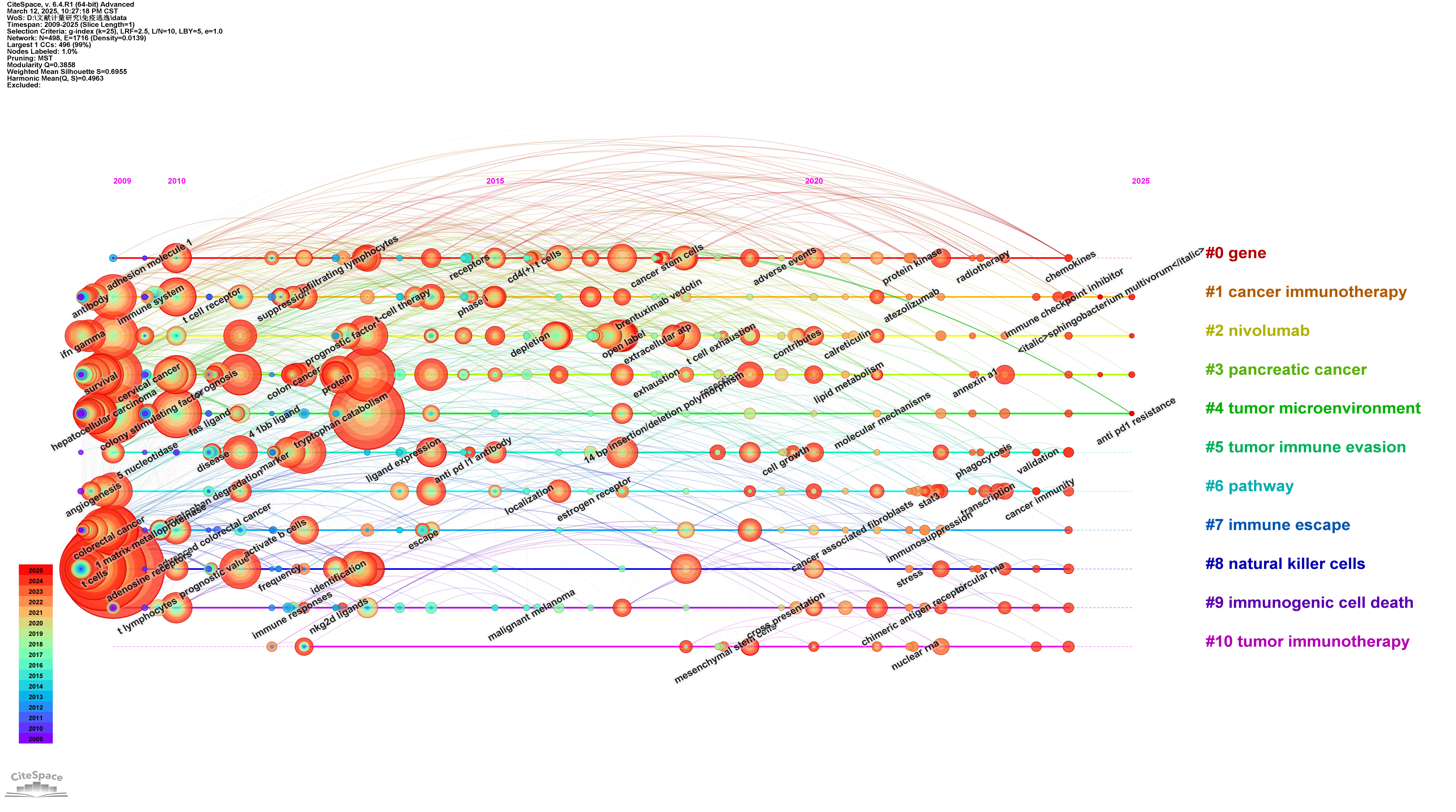

Supplement: Supplementary file 1 [file DataSheet1.zip › supplementary/Figure 10(E).jpg]

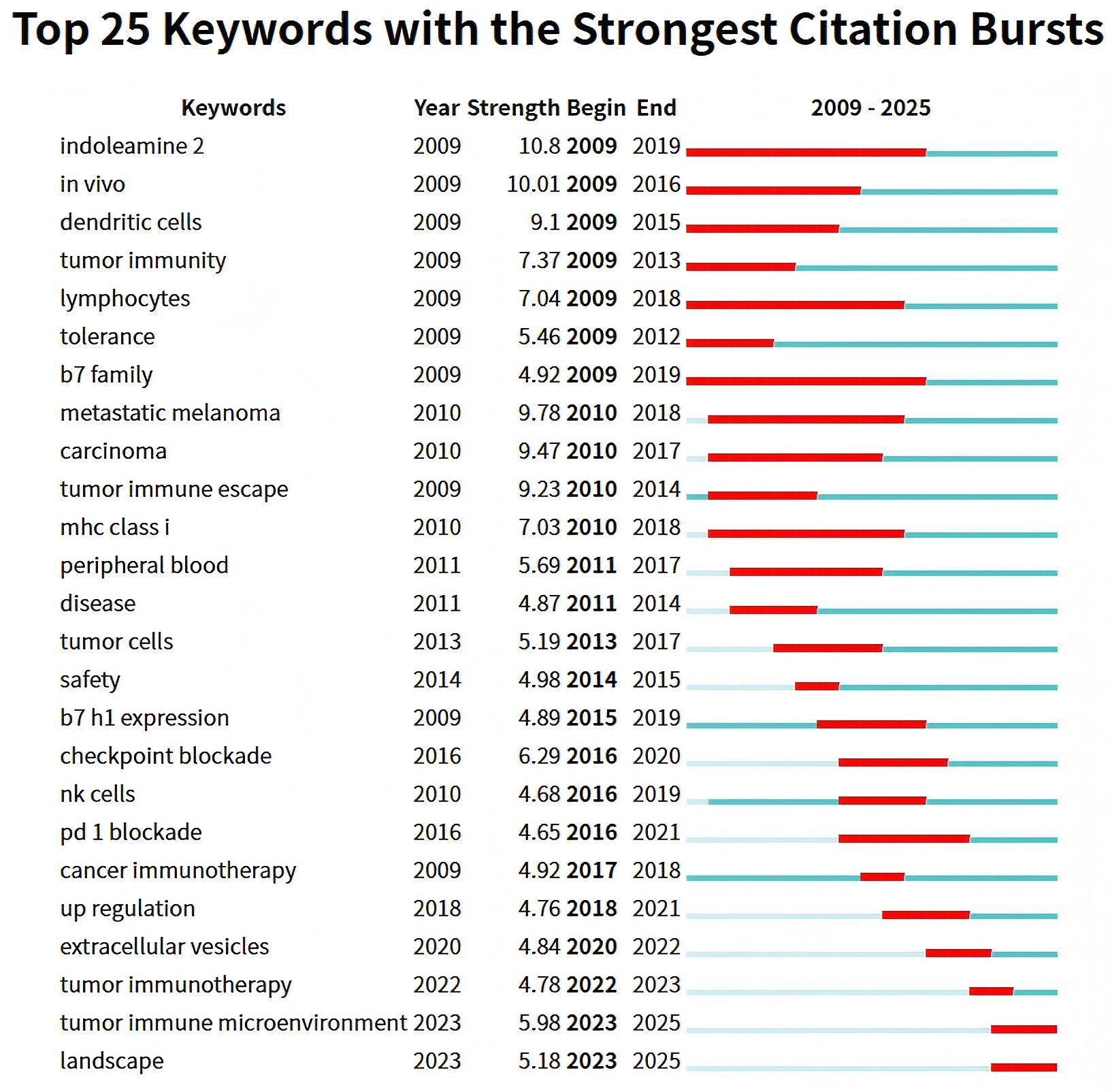

Supplement: Supplementary file 1 [file DataSheet1.zip › supplementary/Figure 10D.jpg]
